# Antibacterial and Anticancer Activity and Untargeted Secondary Metabolite Profiling of Crude Bacterial Endophyte Extracts from *Crinum macowanii* Baker Leaves

**DOI:** 10.1155/2020/8839490

**Published:** 2020-12-10

**Authors:** Tendani E. Sebola, Nkemdinma C. Uche-Okereafor, Lukhanyo Mekuto, Maya Mellisa Makatini, Ezekiel Green, Vuyo Mavumengwana

**Affiliations:** ^1^Department of Biotechnology and Food Technology, Faculty of Science, University of Johannesburg, Doornfontein Campus, Johannesburg, South Africa; ^2^Department of Chemical Engineering, Faculty of Engineering and the Built Environment, University of Johannesburg, Doornfontein Campus, Johannesburg, South Africa; ^3^Molecular Sciences Institute, School of Chemistry, University of the Witwatersrand, Johannesburg, South Africa; ^4^DST-NRF Centre of Excellence for Biomedical Tuberculosis Research, South African Medical Research Council Centre for Tuberculosis Research, Division of Molecular Biology and Human Genetics, Faculty of Medicine and Health Sciences, Stellenbosch University, Tygerberg Campus, Cape Town, South Africa

## Abstract

This study isolated and identified endophytic bacteria from the leaves of *Crinum macowanii* and investigated the potential of the bacterial endophyte extracts as antibacterial and anticancer agents and their subsequent secondary metabolites. Ethyl acetate extracts from the endophytes and the leaves (methanol: dichloromethane (1 : 1)) were used for antibacterial activity against selected pathogenic bacterial strains by using the broth microdilution method. The anticancer activity against the U87MG glioblastoma and A549 lung carcinoma cells was determined by the MTS (3-(4,5-dimethylthiazol-2-yl)-5-(3-carboxymethoxy-phenyl)-2-(4-sulfophenyl)-2H-tetrazolium) assay. Bacterial endophytes that were successfully isolated from *C. macowanii* leaves include *Raoultella ornithinolytica*, *Acinetobacter guillouiae*, *Pseudomonas* sp., *Pseudomonas palleroniana*, *Pseudomonas putida*, *Bacillus safensis*, *Enterobacter asburiae*, *Pseudomonas cichorii*, and *Arthrobacter pascens*. *Pseudomonas cichorii* exhibited broad antibacterial activity against both Gram-negative and Gram-positive pathogenic bacteria while *Arthrobacter pascens* displayed the least MIC of 0.0625 mg/mL. *Bacillus safensis* crude extracts were the only sample that showed notable cell reduction of 50% against A549 lung carcinoma cells at a concentration of 100 *μ*g/mL. Metabolite profiling of *Bacillus safensis*, *Pseudomonas cichorii*, and *Arthrobacter pascens* crude extracts revealed the presence of known antibacterial and/or anticancer agents such as lycorine (1), angustine (2), crinamidine (3), vasicinol (4), and powelline. It can be concluded that the crude bacterial endophyte extracts obtained from *C. macowanii* leaves can biosynthesize bioactive compounds and can be bioprospected for medical application into antibacterial and anticancer agents.

## 1. Introduction

The emergence of infectious diseases worldwide due to bacteria and viruses still poses a serious public health concern, claiming the lives of half a million people a year and amounting to 25% of the total deaths worldwide [1]. Even with the discovery and production of new and improved antibiotics, the resistance of pathogenic microorganisms to drugs has increased enormously [2]. Ventola [3] indicated that the causes of antibiotic resistance include overuse, inappropriate prescribing, and extensive agricultural use. There is, therefore, an imminent need to discover and develop new drugs to combat antimicrobial resistance [4]. The World Health Organization (WHO) has declared that antibiotic resistance is a global public health concern, claiming that there are at least 700,000 annual deaths globally [5, 6]. Novel therapies ought to be discovered to combat antimicrobial resistance [7]. Bacterial infection attributes about 15% of cancers worldwide and, therefore, is a serious health concern [8].

In South Africa, brain cancer seems to be more prevalent in males (0.57%) than females (0.38%) [9]. Gliomas are common primary central nervous system (CNS) tumors mostly affecting the brain [10]. Glioblastomas are aggressive cancers with poor prognosis and an average patient survival of 18 months [11]. In South Africa, lung cancer is more common in males with 4.91% and females with 2.52% [9]. Lung cancer has been deemed as one of the most prevalent cancers in the developed world and has a very poor survival rate since most patients are diagnosed at a stage when curative treatment is impossible. It is one of the hardest cancers to diagnose [12]. The WHO has ranked cancer as the first leading cause of death globally to people below the age of 70 years, with an estimate of 18.1 million new cancer cases and 9.6 million deaths from cancer in the year 2018 [13]. Kumar et al. [14] described that discovery and development of chemotherapeutic agents are vital in the treatment of cancer, since currently used therapies are ineffective and have side effects, and many important anticancer drugs, such as camptothecin, penochalasin A, and chaetoglobosin E, have been isolated from endophytes. Therefore, further investigations on bacterial endophytes have to be conducted.

Endophytes have been reported from different plant species and plant parts, in various geographical locations and diverse environmental conditions [15]. Endophytes inhabit their host plants; they improve drought tolerance and produce protective compounds which protect host plants from biotic and abiotic factors [16]. Furthermore, Dembitsky [17] reported that endophytes produce bioactive secondary metabolites, such as alkaloids and lactones that display antimicrobial and anticancer properties. These bioactive compounds could be explored further for medical, agricultural, and pharmaceuticals use [18]. Endophytes inhabit unique biological niches growing in uncommon environments, and their isolation and identification are vital for further exploration [19, 20].

*Crinum macowanii* Baker is a medicinal plant native to east, central, and southern Africa [21]. The bulbs, leaves, and roots of *C. macowanii* possess medicinal properties and have been used traditionally to treat or manage animal and human diseases [20, 21]. The different plant parts are used for chest pains, diarrhea, tuberculosis, and stimulate milk production in cattle and, thus, are overexploited and overharvested for medicinal purposes [21]. Biological activities of secondary metabolites are attributed to the components working in a mixture of compounds (synergy) as compared to when in isolation [22]; therefore, metabolite fingerprinting of crude endophytes extracts is vital in drug discovery. Morare et al. [24] and Sebola et al. [25] reported on the isolation of bacterial endophytes from the bulbs and the antibacterial activities of their crude extracts, leaving bacterial endophytes isolated from the leaves unexplored. Mixtures of natural extracts are effective in the search for new drugs since they reduce drug-resistant phenotype and hence this study was conducted [26].

The main aim of this study was to isolate and identify bacterial endophytes from *Crinum macowanii* leaves and to explore the role of endophytic crude extracts as potential antibacterial and anticancer therapeutic agents and further profiling of the secondary metabolite produced by the isolated endophytes as a means to halt the overharvesting of *Crinum macowanii* leaves.

## 2. Materials and Methods

### 2.1. Sample Collection

The collection of the leaves was done according to a method described by Sebola et al. [25], where fresh, healthy *C. macowanii* leaves showing no apparent symptoms of disease or herbivore damage were collected from the Walter Sisulu National Botanical Garden (Roodepoort, Gauteng, South Africa, 26°05′10.4″S 27°50′41.5″E). After collection, the samples were placed in sterile polyethylene bags and transferred to the laboratory at 4°C before being thoroughly washed with sterile distilled water and used within hours of harvesting.

### 2.2. Isolation of Bacterial Endophytes

Bacterial endophytes were isolated from the leaves of the plant by a method described by Jasim et al. [27] and Sebola et al. [25] with minor modifications. Briefly, the leaves were cut into small pieces of about 10 cm using a sterile pair of scissors. The cut leaves were treated with 5% Tween 20 (Sigma-Aldrich, South Africa) (enough to cover the plant material) and vigorously shaken for 5 minutes. Tween 20 was removed by rinsing several times with sterile distilled water, followed by disinfection with 50 mL of 70% ethanol for 1 minute. Traces of the ethanol were removed by rinsing with sterile distilled water 5 times. The sample was then treated with 1% sodium hypochlorite (NaClO) for 10 minutes and again rinsed five times with sterile distilled water. The last rinse was used as a control, and 100 *μ*L of this was plated on Potato Dextrose agar (PDA) (HiMedia, USA) and Nutrient Agar (NA) (Oxoid, USA). The sample was then macerated in sterilized phosphate-buffered saline (PBS). The macerated sample was serially diluted up to 10^−3^ dilution, and each dilution was inoculated (using a spread plate method) in triplicate on nutrient agar. The NA plates were incubated at 30°C (IncoTherm, Labotec, Johannesburg, South Africa). Growth was monitored periodically for 5 days. The effectiveness of the sterilization was monitored on the wash control plate, with growth indicating poor sterilization. Under such circumstances, the plates for the plant part were discarded and the sterilization was repeated. Distinct colonies were selected and subcultured on nutrient agar to obtain pure isolates. Pure bacterial isolates were preserved in 50% glycerol in a ratio of 500 *μ*L glycerol : 500 *μ*L overnight broth culture and kept at −80°C.

### 2.3. Morphological Identification of Endophytic Bacteria

#### 2.3.1. Gram Staining

To determine the shape and Gram stain reaction, a method described by Sandle [28] was used. Pure colonies were subjected to Gram staining to establish morphological characteristics such as shape and Gram stain reaction. Gram stain slides were observed using a compound bright-field microscope (OLYMPUS CH20BIMF200) with 1000x magnification.

### 2.4. Molecular Identification

#### 2.4.1. Genomic DNA Extraction, Polymerase Chain Reaction, and Sequencing

The 16S rRNA gene of the bacterial endophyte was amplified according to a method described by Kuklinsky-Sobral et al. [28]. DNA extraction was done using a ZR Fungal/Bacterial Kit™ (Zymo Research, catalog NO R2014) according to the manufacturer's instructions. Polymerase chain reaction (PCR) was done to amplify the 16S rRNA gene of each bacterial endophyte with the primers 16S-27F: 5′-AGAGTTTGATCMTGGCTCAG-3′ and 16S-1492R: 5′-CGGTTACCTTGTTACGACTT-3′, using DreamTaq™ DNA polymerase (Thermo Scientific™). PCR products were gel extracted (Zymo Research, Zymoclean™ Gel DNA Recovery Kit) and sequenced in the forward and reverse directions on the ABI PRISM™ 3500xl Genetic Analyzer. The sequencing was performed at Inqaba Biotechnical Industries (Pty) Ltd., Pretoria, South Africa. The PCR products were cleaned with ExoSAP-it™ following the manufacturer's recommendations. Purified sequencing products (Zymo Research, ZR-96 DNA Sequencing Clean-up Kit™) were analyzed using CLC Main Workbench 7, followed by a BLAST search (NCBI).

### 2.5. Phylogenetic Analysis

The obtained sequences were screened for chimeras using DECIPHER23 and subjected to BLAST analysis using the National Center for Biotechnology Information (NCBI) database against the 16S rDNA sequence database (bacteria and archaea) to identify the closest bacterial species. Bacterial species with 98–100% similarities were selected for phylogenetic analysis. Alignments of nucleotide sequences were performed using MUSCLE with default options. The positions containing gaps or missing nucleotide data were eliminated. Phylogenetic trees were constructed using a Neighbor-Joining (NJ) method (Saitou and Nei, 1987) based on the Tamura-Nei model [29]. A total of 1000 replications were used for bootstrap testing. All branches with greater than 50% bootstraps were considered to be significant [31]. All evolutionary analyses were conducted in MEGA 7.0 [31]. The 16S rRNA gene sequences of bacterial isolates identified in the study were deposited in GenBank (https://www.ncbi.nlm.nih.gov/genbank/) with the accession numbers as stated in Table 1. The assigned names of the bacterial isolates were based on the BLAST homology percentages as well as the phylogenetic results.

### 2.6. Extraction of Crude Extracts from *C. macowanii* Leaves

The extraction of secondary metabolites from the leaves was carried out using the method previously described by Yadav and Agarwala [32] and Sebola et al. [25]. *C. macowanii* leaves were washed, cut into small pieces, and air-dried at room temperature. The dried plant material was blended into a fine powder using a commercial blender. 150 g of the prepared plant material was mixed with 2 L of a 50 : 50 methanol : dichloromethane solution. This was allowed to shake for 3 days on a platform shaker (Amerex Gyromax, Temecula, CA, USA) at 200°rcf. The solution was filtered through Whatman No. 1 filter paper; the filtrate was evaporated on a rotatory evaporator and allowed to air dry in a desiccator.

### 2.7. Extraction of Crude Extracts from Bacterial Endophytes

The extraction of crude extracts from each isolated endophytic bacterium was carried out using the method previously described by Sebola et al. [25]. Briefly, LB broth (1 L) was prepared in 2 L of broth and was measured into a 4 L Erlenmeyer flask leaving room for aeration and autoclaved at 121°C for 15 min. Each 4 L flask was inoculated with one of the endophytic bacteria as listed in Table 1, shaken at 200°rcf, and incubated at 30°C, an ideal temperature for the growth of the endophytes [33]. After 7 days of cultivation, sterile XAD-7- HP resin (20 g/L) (Sigma, Johannesburg, South Africa, BCBR6696V) was added to the culture for 2 h, shaken at 200 rcf. The resin was filtered through cheesecloth and washed three times with 300 mL of acetone for each wash. The acetone soluble fraction was concentrated using a rotary evaporator, and a dark yellowish viscous extract was obtained, which was transferred into a measuring cylinder. Depending on the volume, ethyl acetate was added in a ratio of 1 : 1 (v/v). The mixture was vigorously shaken for about 10 min, decanted into a separating funnel, and allowed to separate and each phase is collected in a conical flask. This process was repeated until the dark yellowish viscous liquid obtained after removing the acetone became a very light-yellow liquid. The ethyl acetate fraction was evaporated using a rotary evaporator, and the brown extract obtained was stored in an amber bottle in a cool dry place until the analysis was done. The light-yellow liquid was evaporated, and no reasonable extract or further analysis was done on this substance. The brown crude endophyte extracts were used for antibacterial and anticancer assays and metabolite fingerprinting.

### 2.8. Antibacterial Analysis of *Crinum macowanii* Bulbs Crude Extracts and Endophytic Bacterial Crude Secondary Metabolite Extracts

The evaluation of the antimicrobial activity of the crude secondary metabolite extract was performed using the minimum inhibition concentration (MIC) method as previously described by Sebola et al., Sebola et al., and Andrews, [25, 34, 35]. Eleven pathogenic bacterial species, namely, *Bacillus cereus* (ATCC10876), *Bacillus subtilis* (ATCC19659), *Streptococcus epidermidis* (ATCC14990), *Staphylococcus aureus* (ATCC25923), *M. smegmatis* (ATCC21293), *Mycobacterium marinum* (ATCC927), *Enterobacter aerogenes* (ATTC13048), *Escherichia coli* (ATCC10536), *Klebsiella pneumonia* (ATCC10031), *Proteus vulgaris* (ATCC 33420), and *Proteus aeruginosa* (ATCC10145) were used. The antibiotic Streptomycin was used as the positive control and was prepared by weighing 0.032 mg in 1 mL of sterile distilled water while 0.1% DMSO was used as a negative control.

#### 2.8.1. Sample Preparation

The crude leave extract and crude endophytic extracts were weighed separately into empty autoclaved McCartney bottles to ensure sterility. A minimal amount of dimethyl sulfoxide (DMSO) (0.1%) was used to dissolve the crude extracts, and Mueller-Hinton (MH) broth was added to bring the volume of the dissolved crude extract to a concentration of 32 mg/mL as the stock solution.

#### 2.8.2. Microtiter Plate Assay

Serial dilutions were carried out using the MH broth from 16 mg/mL down to 0.031 mg/mL, which was the lowest inhibition observed. The experiment was carried out in five repeats using a 96-well microtiter plate. The outer wells of the plate were filled with sterile distilled water (sdH_2_O). The inoculum (100 *μ*L) was added to each well that did not contain the sdH_2_O. The diluted crude extract samples (100 *μ*L) were added in five wells horizontally, and the concentrations decreased in a vertical order from 16 mg/mL down to 0.031 mg/mL. The plates were covered and incubated overnight at 37°C. After incubation, 10 *μ*L of 0.02% (w/v) Resazurin sodium salt dye solution was added to the wells, and the resulting solution was incubated for another two hours. On reduction, resazurin changes color from blue to pink to clear as oxygen becomes limited within the medium, indicating metabolism and the viability of bacterial cells, as well as no effect of the crude extracts on the bacteria. Any well with a known concentration showing a slight color change was used as MIC. The wells were visually inspected for color changes.

### 2.9. Anticancer Assays

The evaluation of the anticancer activity of the crude secondary metabolite extract was performed using the MTS (3-(4, 5-dimethylthiazol-2-yl)-5-(3-carboxymethoxy-phenyl)-2-(4-sulfophenyl)-2H-tetrazolium) assay as previously described by Sebola et al. [25]. A stock solution of 200 *μ*g/mL of all crude extracts (leave crude extracts and endophytic crude extract) was prepared in 0.1% DMSO and sonicated. Serial dilutions were done according to [36, 37]. Briefly, dilutions were carried out using growth media from 100 *μ*g/mL to 3.13 *μ*g/mL. MTS (3-(4, 5-dimethylthiazol-2-yl)-5-(3-carboxymethoxy-phenyl)-2-(4-sulfophenyl)-2H-tetrazolium) *in vitro* cancer cytotoxicity assay was carried out to determine a change in cell viability through the use of a color change. The MTS compound (yellow) is metabolized by viable cells to form a dark purple-colored compound, while dead cells turn the color of the MTS compound pink. The samples were run in duplicate across three plates (*n* = 6), and the average values obtained were reported. The U87MG (glioblastoma) cells and A549 (lung carcinoma) cells were grown using normal tissue culture techniques using Dulbecco's Modified Eagle Medium (Merk, Johannesburg, SA) supplemented with 15% fetal bovine serum (FBS) (Merck, Johannesburg, SA). The cells (1 × 10^5^ cells/ml) were incubated in 96-well plates at 37°C overnight, with the subsequent addition of the crude bulb extracts and crude endophytic extracts, in concentrations of 100 *μ*g/mL, 50.0 *μ*g/mL, 25.0 *μ*g/mL, 12.5 *μ*g/mL, 6.25 *μ*g/mL, 3.125 *μ*g/mL, and 0 *μ*g/mL. The cells were left to incubate for 4 days, whereupon MTS (5 *μ*L) (Promega, Madison, WI, USA) was added to the cells. The absorbance values were measured at 490 nm after 1 hr, 2 hr, and 4 hr incubation periods. Cell viability was then calculated using the formula

(1) $$\begin{array}{c} \%\text{ Cell viability}=\frac{\left(E_{\text{a}}-B_{\text{a}}\right)}{\left(C_{\text{a}}-B_{\text{a}}\right)}\times 100, \end{array}$$

where *E*_a_ is the absorbance of the extract, *B*_a_ is the absorbance of the blank, and *C*_a_ is the absorbance of the control [39]. The positive control used for all conducted tests was auranofin, as it is able to inhibit thioredoxin reductase as well as the ubiquitin–proteasome system (UPS) by targeting proteasome-associated deubiquitinase, thus inducing lung cancer cell apoptosis by selenocystine [39–41].

### 2.10. LC-QTOF-MS Analysis

#### 2.10.1. Instrumentation

Secondary metabolites of the crude leave extracts and crude endophytes extracts were identified using an LC-QTOF system with a Dionex UltiMate 3000 UHPLC (Thermo Scientiﬁc, Darmstadt, Germany) coupled to a Compact™ QTOF (Bruker Daltonics, Bremen, Germany) that uses an electrospray ionization (ESI) interface, following a modified method by Hoffman et al., Changwa et al., Want, Tapfuma et al., and Tapfuma et al. [42–46]. The instrument parameters used in this study are listed in Table 2. Instrument operation control and acquisition was done using HyStar software version 2.1 (Thermo Scientific, Darmstadt, Germany). The analytical run was set at 40 minutes.

The gradient flow used for the mobile phase is listed in Table 3.

#### 2.10.2. Data Processing

Spectral data processing was performed on Bruker Compass DataAnalysis software version 4.3 (Bruker Daltonics, Brem Hub, California, USA). MetFrag web tool version 2.1 software (Git Hub, California, USA) was used to compare fragment patterns of fragmented ions with those from compound databases, namely, PubChem, ChemSpider, and KEGG [48]. Additional databases that were used include METLIN (Scripps Research, California, USA) and KNapSAcK (Kanaya Laboratory, Japan) [49]. Blank containing methanol for plant extracts and NB for bacteria endophytes were also analyzed in the same conditions. Comparison of the two base chromatograms (from the crude extracts and endophyte extracts and the controls) allowed for filtering out impurities from the growth medium [45, 46].

## 3. Results and Discussion

### 3.1. Molecular and Morphological Identification of Bacterial Endophytes from *C. macowanii* leaves

Endophytes inhabit unique biological niches growing in unusual environments. Their isolation and identification are vital for further exploration [49, 50]. In this study, a total of 9 bacterial endophytes were isolated and characterized from the leaves of *C. macowanii*. as seen in Table 1.

Seven Gram-negative bacteria were observed. Phenotype diversity of the endophyte contributes to the Gram reaction and colony morphology of the endophytes [51] and hence the diverse endosphere, while the growth rate and size of the host plant also have an effect on the diverse endophytic community [52]. The endophytic community of plants is influenced by the age of the host plant, geographic location, and abiotic factors such as temperature [52, 53]. This would explain the diversity of the bacterial endophytes isolated.

**Figure 1:**
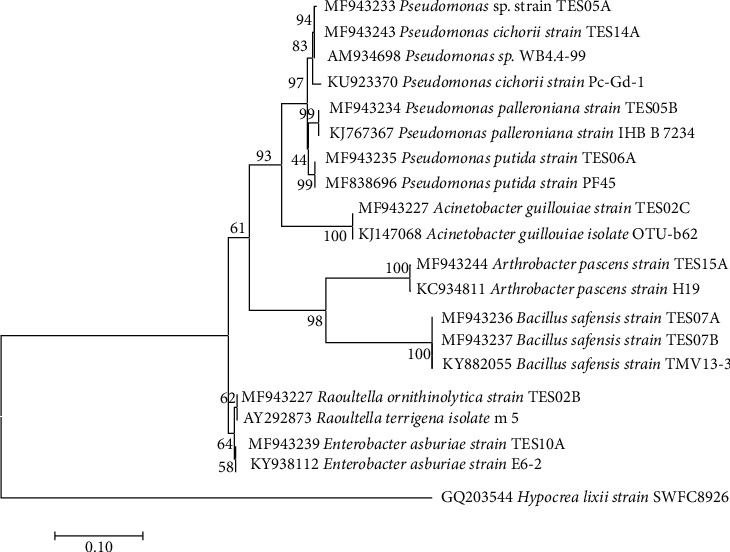
Neighbor-joining phylogenetic tree of 16S rRNA gene sequences of endophytes isolated from *C. macowanii* leaves showing the relationship with other similar species selected from GenBank.

Bacterial genera including *Pseudomonas*, *Bacillus*, and *Burkholderia* have been isolated as endophytes from leaves of medicinal plants [54]. These bacteria genera predominate in medicinal plants, as they assist the host plant in mineral nutrient composition and protection against abiotic and biotic stresses [55]. The BLAST search conducted of the results of the 16S rDNA gene sequence revealed that the isolated endophytes belong to bacterial genera, such as *Raoultella*, *Acinetobacter, Pseudomonas, Bacillus, Enterobacter*, and *Arthrobacter*, as seen in Figure 1. This supports our observed results.

*Pseudomonas* are common bacteria associated with plants and have been isolated from a number of plant species and tissues. They display a positive effect on host plant growth such as reducing drought stress and producing plant hormones such as 1-aminocyclopropane-1-carboxylic acid (ACC) and Indole-3-acetic acid (IAA) and acting as biocontrol agents [26, 49, 56, 57]. Our isolate TES05A showed 94% similarity with *Pseudomonas* sp. WB4.4-99. Endophytic isolate TES14A showed 83% similarity with *Pseudomonas cichorii* Pc-Gd-1. Endophytic *Pseudomonas cichorii* has been isolated from potato cultivar [58]. Isolate TES06A displayed a 99% similarity with *Pseudomonas putida* PF45. Endophytic *Pseudomonas putida* have been isolated from mango orchard [59]. Isolate TES05B showed 99% similarity with *Pseudomonas palleroniana* IHB B 7234. Endophyte *Pseudomonas palleroniana* has been isolated from bananas and is reported to fix free nitrogen, solubilize phosphates, and produce siderophores *in vitro* [60].

TES02C displayed a sequence identity of 100% to *Acinetobacter guillouiae* OTU-b62. Different *Acinetobacter* species have been isolated from potato cultivars [58]. TES15A isolates revealed 100% to *Arthrobacter pascens* H19. *Arthrobacter* spp. has been isolated from ethnomedicinal plants in Southern India [61]. *Bacillus* endophytes have been isolated from sunflower, potatoes, and cotton and assist host plants in phosphate solubilization and auxin production [56]. Our endophytic isolates (TES07A and TES07B) displayed 100% similarity to *Bacillus safensis* TMV13-3. *Bacillus* spp. Isolates TES02B had a similarity of 62% to *Raoultella terrigena m* 5. Endophytic *Raoultella ornithinolytica* has been isolated from mountain-cultivated ginseng plants [62]. A 64% similarity was observed between our isolate TES10A and *Enterobacter asburiae* E6 – 2. Endophytic *Enterobacter asburiae* has been isolated from date palm and promotes plant growth [63].

To the best of our knowledge, this is the first report on the isolation of *Arthrobacter pascens* and *Enterobacter asburiae* from *C. macowanii*.

### 3.2. Antibacterial Evaluation of Crude Bacteria Endophyte Extracts from the Leaves

The lowest MIC (0.0625 mg/mL) was observed from *Arthrobacter pascens* crude extract and crude extracts against *B. subtilis,* respectively. The crude extracts of most of the endophytes showed MIC values below 1.00 mg/mL. The leave crude extracts displayed noteworthy activity against both Gram-positive and Gram-negative bacteria as seen in Table 4.

*C. macowanii* leaves have been used traditionally to cleanse the blood, treat coughs, kidney, and bladder diseases in humans and animals, and also t treat coughs and diarrhea [20]. The results obtained in this study indicate the inhibition of *B. subtilis*, *M. smegmatis,* and *P. vulgaris* at 0.500 mg/mL, and *S. aureus*, *E. coli,* and *K. pneumonia* were inhibited at 0.250 mg/mL and *S. epidermidis* and *P. aeruginosa* at 1.00 mg/mL and 0.125 mg/mL, and respectively.

These data provide scientific justification for the ethnomedicinal uses of the leaves, as the inhibited bacteria are the main causative agents of the ailments the leaves are used to treat. *S. aureus*, *E. coli*, and *K. pneumoniae* were inhibited by a final concentration of 250 *μ*g·mL^−1^ by alkaloids such as crinine, cherylline, crinamidine, 3-O-acetylhamayne, and bulbispermine which have been isolated from *C. macowanii* leaves [64]. To the best of our knowledge, this study is the first to report on the extraction of crude extract from leaves of *C. macowanii* and its antibacterial activity.

The cultivation of plants to obtain bioactive compounds has led to drawbacks, such as overharvesting of plants to obtain bioactive compounds, different environmental conditions tend to produce low yields, and total synthesis and semisynthesis are challenging due to complex structures [65]. A number of endophytic microorganisms have produced anticancer, antimicrobial, antidiabetic, insecticidal, and immunosuppressive compounds [66]. Plants growing in a variety of places possibly harbor endophytes with novel natural products [66, 67].

*Raoultella ornithinolytica* crude extract had MIC values ranging from 0.250 to 16 mg/mL, with the most significant inhibition observed for *K. pneumonia*, *E. coli,* and *M. marinum* at concentrations of 0.500 mg/mL, and *P. aeruginosa* was inhibited at concentrations of 0.250 mg/mL. Microcin genes have been reported to be present on *Raoultella ornithinolytica* [62] and microcins are antibacterial peptides produced by *Enterobacteria* [68]. This could explain the observed results. *Acinetobacter guillouiae* crude extract showed MIC values of between 0.500 and 16 mg/mL. The crude extract showed activity against *M. marinum* at 0.500 mg/mL.

*Bacillus safensis* crude extract showed MIC values of between 0.125 and >16 mg/mL. The crude extract showed activity against *B. subtilis*, *M. marinum,* and *E. coli* at concentrations of 0.125 mg/mL, 0.500 mg/mL, and 0.250 mg/mL, respectively. Crude endophytic extracts of *Bacillus safensis* isolated from *Ophioglossum reticulatum* L. displayed antibacterial activity against *Staphylococcus aureus* and *Escherichia coli* [69]. This is in agreement with the obtained results.

*Enterobacter asburiae* crude extract showed MIC values of between 0.125 and 16 mg/mL. The crude extract showed activity against *M. smegmatis* and *E. coli* at concentrations of 0.500 mg/mL and *M. marinum* and *K. pneumonia* at concentrations of 0.125 mg/mL. Endophytic crude extracts of *Enterobacter asburiae* displayed antibacterial activity against *K. pneumoniae*, *E. coli*, *S. aureus*, and *B. cereus*. *Enterobacter* strains [70] have been reported to produce antibacterial lipopeptides with a broad activity [71]. This supports the obtained results.

*Arthrobacter pascens* crude extract showed MIC values of between 0.0625 and >16 mg/mL. The most active inhibition was against *B. subtilis* at 0.0625 mg/mL. The crude extract showed activity against *S. aureus* and *E. aerogenes* at concentrations of 1.00 mg/mL. Arthrobacilin, an antibacterial compound produced by *Arthrobacter* spp., showed inhibition against *S. aureus* [72, 73]. This could explain the antibacterial activity observed.

*Pseudomonas* sp. crude extract showed MIC values of between 0.0625 and >16 mg/mL. The most active inhibition was against *M. marinum* at 0.0625 mg/mL. Mupirocin produced by *Pseudomonas* strains has been reported to possess antibacterial activity [74]. *Pseudomonas palleroniana* crude extract showed MIC values of between 0.250 and >16 mg/mL. The most active inhibition was against *P. vulgaris* at 0.250 mg/mL. The crude extract showed activity against *E. aerogenes* and *P. aeruginosa* at concentrations of 1.00 mg/mL and *M. marinum* and *K. pneumonia* at concentrations of 0.500 mg/mL. Endophytic crude extracts from *Pseudomonas palleroniana* were reported to inhibit *Escherichia coli* and *Staphylococcus aureus* [50]. Pyoluteorin produced by *Pseudomonas palleroniana* strains has been reported to contain antibacterial activity [75]. *Pseudomonas putida* crude extract showed MIC values of between 1.00 and >16 mg/mL. The most active inhibition was against *P. aeruginosa* at 1.00 mg/mL. Antibiotics pyoluteorin, phenazine-1-carboxamide, and phenazine-1-carboxylic acid were produced by *Pseudomonas putida* strains [75]. This could explain the antibacterial activity observed. *Pseudomonas cichorii* crude extract showed MIC values of between 0.125 and 16 mg/mL. The crude extract showed activity against *E. coli* and *P. vulgaris* at concentrations of 1.00 mg/mL, *M. smegmatis* and *P. aeruginosa* at concentrations of 0.125 mg/mL, *B. subtilis* at 0.250 mg/mL, and *K. pneumonia* at 0.500 mg/mL. To the best of our knowledge, this is the first report on the antibacterial activity of crude endophyte extracts from *Pseudomonas cichorii*.

Cos et al. [76] state that a concentration of <0.1 mg/mL for a crude sample is the ideal concentration for anti-infective bioassays whereas [77, 78] recommend that crude samples with a concentration of 1.00 mg/mL and ≤100 *μ*g/ml (0.100 mg/ml) and ≤625 *μ*g/ml are considered to be very significant and moderately significant and therefore noteworthy for minimal inhibitory concentration. Stringent endpoints for anti-infective bioassays ought to be set to prevent false results and confusion, taking into consideration the sensitivity of extracts and test microorganisms, extraction methods, and solvents used [66, 67].

It was observed from the results that crude endophyte extract from *Pseudomonas* sp. and *Arthrobacter pascens* obtained from *C. macowanii* leaves had noteworthy antibacterial activity against the pathogenic bacteria used in this study and can be used as antibacterial agents against and *M. marinum* and *B. subtilis* infections, respectively.

### 3.3. Anticancer Evaluation of Crude Bacteria Endophyte Extracts from the Leaves against Resistance Cancer Cell Lines

Secondary metabolites produced by endophytic microorganisms' act as anticancer agents and display significant potential in medical and veterinary treatments [79, 80]. Anticancer agents paclitaxel and podophyllotoxin have been isolated from endophytic microorganisms [81].

#### 3.3.1. Anticancer Evaluation of Crude Bacteria Endophyte Extracts from the Leaves against A549 Lung Carcinoma Cells

**Figure 2:**
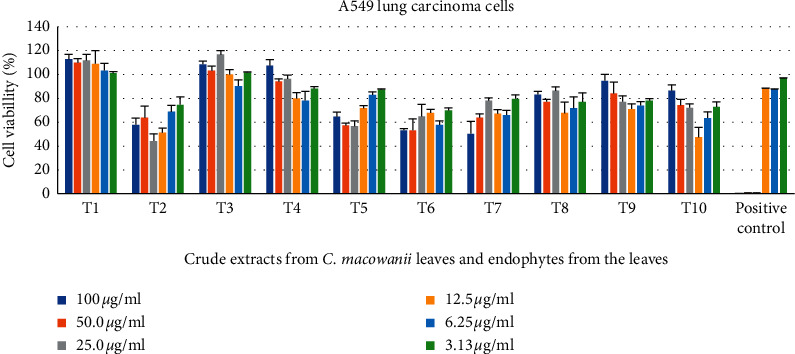
Cytotoxic activity of endophytic-derived secondary metabolites and crude extracts on A549 lung carcinoma cells tested at different concentrations ranging from 100 to 3.13 *μ*g/mL. The positive control used was auranofin. T1 = *C. macowanii* leaves, T2 = *Raoultella ornithinolytica*, T3 = *Acinetobacter guillouiae*, T4 = *Pseudomonas* sp., T5 = *Pseudomonas palleroniana*, T6 = *Pseudomonas putida*, T7 = *Bacillus safensis*, T8 = *Enterobacter asburiae*, T9 = *Pseudomonas cichorii,* T10 = *Arthrobacter pascens*.

*Pseudomonas putida* and *Bacillus safensis* crude extracts showed a 47% and 50% cell reduction, respectively, against lung carcinoma cells at a concentration of 100 *μ*g/mL as seen in Figure 2.

#### 3.3.2. Anticancer Evaluation of Crude Bacteria Endophyte Extracts from the Leaves against UMG87 Glioblastoma Cells

**Figure 3:**
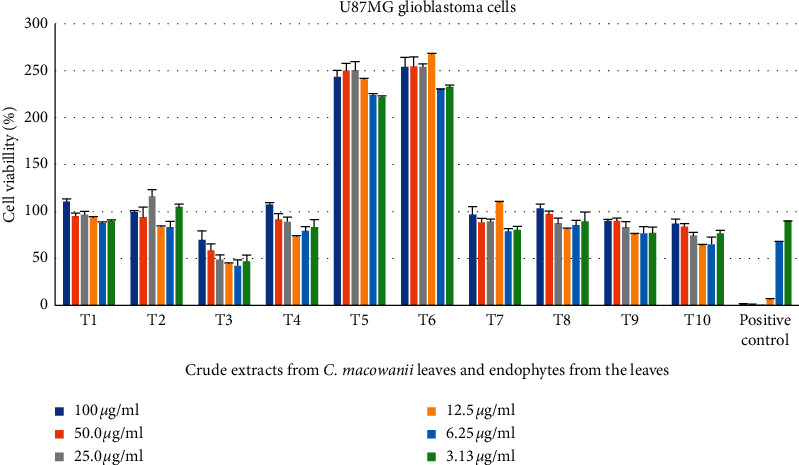
Cytotoxic activity of endophytic-derived secondary metabolites and crude extracts on UMG87 glioblastoma cells tested at different concentrations ranging from 100 to 3.13 *μ*g/mL. The positive control used was auranofin. T1 = *C. macowanii* leaves, T2 = *Raoultella ornithinolytica*, T3 = *Acinetobacter guillouiae*, T4 = *Pseudomonas* sp., T5 = *Pseudomonas palleroniana*, T6 = *Pseudomonas putida*, T7 = *Bacillus safensis*, T8 = *Enterobacter asburiae*, T9 = *Pseudomonas cichorii,* T10 = *Arthrobacter pascens*.

*Acinetobacter guillouiae* crude extracts showed a 42% reduction of UMG87 glioblastoma cells at a concentration of 6.25 *μ*g/mL and *Arthrobacter pascens* crude extracts displayed cell reduction of 37% at a concentration of 12.5 *μ*g/ml as seen in Figure 3.

To the best of our knowledge, this is the first report on the anticancer activity of *C. macowanii* leave crude extracts. No noteworthy activities were observed from the leaves' crude samples against both cell lines used in this study. Bioactive compounds such as lycorine, pretazettine, crinamine, augustine, and galanthamine are noted to appear in *C. macowanii* leaves and have been reported to possess anticancer activity [82–84].

To the best of our knowledge, this is the first report on the anticancer activity of crude endophytes extracts from *Pseudomonas palleroniana*, *Bacillus safensis*, *Enterobacter asburiae*, *Arthrobacter pascens,* and *Pseudomonas cichorii*.

*Acinetobacter guillouiae* crude endophyte extract was the only tested sample that exhibited anticancer against UMG87 glioblastoma cells, with a 31% cell reduction at 100 *μ*g/mL and 53% cell reduction at 3.13 *μ*g/mL posing as a possible anticancer agent against brain cancer.

*Bacillus safensis* crude extracts displayed noteworthy activity against A549 lung carcinoma cells with 50% cell reduction at 100 *μ*g/mL. Crude extracts of *Bacillus safensis* isolated from sea sponges had anticancer activity against HepG2 (hepatocellular carcinoma), HCT (colon carcinoma), and MCF 7 (breast carcinoma) [85]. This could explain the observed results, and crude endophyte extracts from *Bacillus safensis* can be used as an anticancer agent against lung cancer.

Crude endophytic extract of *Enterobacter asburiae, Pseudomonas* sp., *Arthrobacter pascens*, and *Pseudomonas palleroniana* displayed no noteworthy activity against UMG87 glioblastoma cells and A549 lung carcinoma cells. The extracts can be tested on other cancer cell lines to determine their activity. *Pseudomonas* sp. are known to produce anticancer compounds and have been reported to have activity against a number of human cancer cell lines [86, 87]. *Pseudomonas* sp. and *Pseudomonas cichorii* displayed no noteworthy activity against UMG87 glioblastoma cells and A549 lung carcinoma cells. *Pseudomonas palleroniana* crude extracts displayed 36% cell reduction at 100 *μ*g/mL against A549 lung carcinoma cells.

Crude endophyte extracts from *Pseudomonas putida* displayed 47% cell reduction at 100 *μ*g/mL against A549 lung carcinoma cells. *P. putida* TJ151 is able to produce fluorouracil which is a bioactive aromatic compound and it is an anticancer drug [59]. L-methioninase, an enzyme produced by *Pseudomonas putida,* has shown anticancer activity against leukemia cell lines, liver HepG2, breast MCF-7, lung A549, prostate PC3, and colon HCT116 [88, 89]. Methioninase from *P. putida* and 5-fluorouracil work synergistically to inhibit tumor growth and hence the activity observed [90].

Crude endophyte extracts from *Raoultella ornithinolytica* displayed 43% cell reduction at 100 *μ*g/mL against A549 lung carcinoma cells. Protein complex from *R. ornithinolytica* has shown anticancer activity against HeLa cell line, human endometrioid ovarian cancer line (TOV 112D ATCC CRL-11731), and the human breast adenocarcinoma line (T47D ECACC 85102201) resulting in cytopathic effect and reduction in the cell number [91, 92].

Crude endophyte extracts of *Raoultella ornithinolytica*, *Pseudomonas palleroniana*, *Pseudomonas putida,* and *Bacillus safensis* can be further purified and tested for their anticancer activity against other types of the cancer cell line.

### 3.4. Liquid Chromatography Quadrupole Time-of-Flight Mass Spectrometry LC-Q-TOF-MS Analysis

The isolation and characterization of bioactive secondary metabolites help in distinguishing between new and already known bioactive secondary metabolites which help in the development and discovery of new drug leads [93]. Lycorine and powelline were some of the identified secondary metabolites present in the crude extract of *C. macowanii* leaves, *Bacillus safensis*, *Pseudomonas cichorii,* and *Arthrobacter pascens*. These are indicated in Table 5.

Not much work has been done to the leaves, and to the best of our knowledge, this is the first report of the identification of secondary metabolites from leaves of *C. macowanii* and their endophytes using LC-Q-TOF-MS. Endophytes are able to produce similar secondary metabolites as the host plants by exchanging fragments of their genomic DNA with the host plant [103, 104]. These secondary metabolites perform distinct functions such as antibacterial and anticancer activity as performed in this study [105]. Melicopicine is an acridone alkaloid isolated from leaves of *Teclea* and *Zanthoxylum* species [94]. An acridone alkaloid melicopicine has been isolated from *Melicope fareana* [106] and has anthelmintic and antibacterial activities [107]. This would explain the noteworthy antibacterial activity of the crude leave extract observed in this study.

Lycorine is an alkaloid previously isolated from *C. macowanii* [108]. Leaves of *C. macowanii* contain more lycorine as compared to the bulbs and other plant parts such as roots and flowers [109]. Crude endophyte extras of *Bacillus safensis*, *Pseudomonas cichorii*, and *Arthrobacter pascens* displayed the presence of lycorine, and this is not surprising as endophytes can metabolize secondary metabolites from the host plant [104]. Lycorine has been reported to possess antibacterial activity and cytotoxic and antitumor activities [95]. This would explain the observed antibacterial and/or anticancer activity of the crude endophyte extracts.

Angustine is an alkaloid previously isolated from plants of the Rubiaceae and Loganiaceae family [110]. To the best of our knowledge, angustine is being identified for the first time in *C. macowanii* leaves extracts and its isolated endophytes.

Aulicine and 3-O-methyl-epimacowine are crinine-type alkaloids and have been isolated from *Hippeastrum aulicum* Herb. and *Hippeastrum calyptratum* Herb. [110, 111]. Evidente and Kornienko [97] reported on the anticancer properties of these crinine-type alkaloids. This would justify the anti-lung cancer activity of crude *Bacillus safensis* endophyte extracts. Different cancer cell lines can be used to determine the anticancer activity of crude *Pseudomonas cichorii* endophyte extracts.

Crinine-type alkaloid crinamidine has been isolated from different plants of the Amaryllidaceae family [113] and also from the bulbs, flowering stalks, leaves, and roots of *Crinum macowanii* [21]. Crinamidine is found in whole plant parts of the *Crinum* species [114]. Powelline is an alkaloid reported to occur in *C. macowanii* [115] and Ndhlala et al. [115] reported its occurrence from the bulbs. Both these crinine-type alkaloids have been reported to possess antibacterial, antitumor, and anticancer activity [20, 98, 100]. This is not alarming as endophytes can metabolize secondary compounds from the host [105] and display a number of biological activities as the host plant.

Vasicinol is a quinazoline alkaloid from *Adhatoda zeylanica* Medic. [117]. Crude endophyte extracts of *Bacillus safensis*, *Pseudomonas cichorii*, and *Arthrobacter pascens* displayed the presence of this alkaloid; this is not surprising as some quinazoline alkaloids are produced by microbes [118]. Vasicinol can be tested on other resistant pathogenic bacteria to combat antimicrobial resistance, as its antibacterial activity has been reported by Jain et al. [99].

Brefeldin A is a fungal metabolite produced by species of the Ascomycetes [119], and to the best of our knowledge, it is being reported for the first time in bacterial endophytes. Brefeldin A has been reported to possess anticancer activity [101]; different cancer cell lines can be used to determine its anticancer activity with different cell lines.

From the results obtained, varying retention times were observed between the leaves and bacterial endophytes samples, even though the same chromatography conditions were used. Factors such as the affinity of the compounds to the extraction solvents used [120], the change in polarity of the sample being analyzed, and fragments that make up the secondary metabolites detected [121], and the formation of secondary metabolites complexes with extraction solvents used to influence the different retention times observed, as they create a sample matrix [122].

The identified alkaloids lycorine, crinamidine, and powelline are true alkaloids of the Amaryllidaceae family [120, 124], and this supports the obtained results as *Crinum macowanii* belongs to this plant family. The availability of these alkaloids leads to overuse and overharvesting of *C. macowanii* to obtain these bioactive compounds [65, 125]. The bioprospecting of endophyte isolated metabolites could help save the environment since endophytes have been reported to contain similar bioactive compounds as the host plant [66, 126] and in some doing medicinal plants are being conserved and revenue is generated by the bioprospecting of metabolites from endophytes [127].

## 4. Conclusions

The study revealed the presence and cohabitating of endophytic bacteria from leaves of *C. macowanii,* and this has informed us of the microbial community of *C. macowanii*. Crude endophyte extracts displayed notable inhibitory activities against both Gram-positive and Gram-negative bacterial species. Crude extracts endophytes (*Pseudomonas putida* and *Bacillus safensis*) exhibited promising anticancer activity against lung cancer. The identified secondary metabolites from the endophytes have reported biological activities, and this data raises the possibility that the overharvesting of *C. macowanii* for its medicinal properties will be halted. This is a promising lead for drug discovery and bioprospecting. Further extraction of secondary metabolites from endophytes is still needed.

## Figures and Tables

**Table 1:** Endophytic bacteria isolated from *C. macowanii* leaves.

Sample code	Assigned bacterial name	GenBank accession number	Similarity (%)	Gram reaction	Colony morphology (pigmentation, texture, form)
TES 02B	*Raoultella ornithinolytica*	MF943227	99	−Rod	Cream white, moist, circular
TES 02C	*Acinetobacter guillouiae*	MF943228	100	−Rod	Cream white, dry, circular
TES 05A	*Pseudomonas* sp.	MF943233	99	−Rods	White, dry, filamentous
TES 05B	*Pseudomonas palleroniana*	MF943234	100	−Rods	Pale yellow, viscid, filamentous
TES 06A	*Pseudomonas putida*	MF943235	99	−Rods	Milky white, moist, circular
TES 07A	*Bacillus safensis*	MF943236	100	+Rod	Milky white, viscoid, circular
TES 07B	*Bacillus safensis*	MF943237	100	+Rod	White, dry, circular
TES 10A	*Enterobacter asburiae*	MF943239	99	−Rod	Pale yellow, moist, filamentous
TES 14A	*Pseudomonas cichorii*	MF943243	99	−Rods	Yellow, moist, circular
TES 15A	*Arthrobacter pascens*	MF943244	99	+Rods	Cream white, viscid, circular

**Table 2:** Parameters of the LC-QTOF-MS/MS system.

Specification	Setting
Column	Raptor ARC-18 column with dimensions of 2.7 *μ*m (particle size), 2.1 mm (internal diameter), 100 mm (length) and 90 Å (pore size) (Restek, Bellefonte, USA
Injection volume	5 *μ*L
Operating	Reverse phase ultra-high-performance liquid chromatography (RP-UHPLC)
Capillary voltage at	4.5 kV
End plate offset	−500 V
Dry heater nebulizer gas pressure	1.8 bar
Scan	50 to 1300 m/z

**Table 3:** Gradient flow profiles of the mobile phase.

Time (min)	Flow (mL/min)	Solvent A (0.1% formic acid in H_2_O (v/v))	Solvent B (0.1% formic acid in acetonitrile (v/v))
0–2	300	95	5
2–30	300	5	95
30–40	300	95	5

**Table 4:** Antibacterial evaluation of *C. macowanii* crude leave extract and crude endophyte extracts from the leaves.

*Test organism with MIC (mg/mL)*											
Crude extracts	*B. cereus*	*B. subtilis*	*S. epidermidis*	*S. aureus*	*M. smegmatis*	*M. marinum*	*E. aerogenes*	*E. coli*	*K. pneumonia*	*P. vulgaris*	*P. aeruginosa*
T1	2.00	**0.500**	**1.00**	**0.250**	**0.500**	2.00	8.00	**0.250**	**0.250**	**0.500**	**0.125**
T2	8.00	1.00	4.00	16.00	**1.00**	**0.500**	**1.00**	**0.500**	**0.500**	>16.00	**0.250**
T3	4.00	4.00	16.00	8.00	**0.500**	16.00	16.00	8.00	16.00	8.00	16.00
T4	**0.500**	**0.125**	**0.125**	**0.500**	8.00	**0.125**	16.00	>16.00	4.00	16.00	8.00
T5	>16.00	2.00	4.00	4.00	8.00	**0.500**	**1.00**	2.00	**0.500**	**0.250**	**1.00**
T6	>16.00	4.00	8.00	16.00	4.00	>16.00	16.00	>16.00	>16.00	8.00	1.00
T7	>16.00	**0.125**	2.00	>16.00	4.00	**0.500**	16.00	**0.250**	2.00	4.00	>16.00
T8	4.00	16.00	8.00	2.00	**0.500**	**0.125**	8.00	**0.500**	**0.125**	16.00	2.00
T9	8.00	**0.250**	16.00	**1.00**	**0.125**	8.00	4.00	**1.00**	**0.500**	**1.00**	**0.125**
T10	2.00	**0.0625**	4.00	**1.00**	16.00	>16.00	**1.00**	**0.500**	>16.00	2.00	4.00

*Positive control MIC (μg/mL)*											
T11	0.031	0.031	0.062	0.031	0.062	0.062	0.125	0.125	0.125	0.062	0.031

T1 = *C. macowanii* leaves, T2 = *Raoultella ornithinolytica*, T3 = *Acinetobacter guillouiae*, T4 = *Pseudomonas* sp., T5 = *Pseudomonas palleroniana*, T6 = *Pseudomonas putida*, T7 = *Bacillus safensis*, T8 = *Enterobacter asburiae*, T9 = *Pseudomonas cichorii*, T10 = *Arthrobacter pascens*, T11 = Positive control streptomycin.

**Table 5:** LC-Q-TOF-MS analysis of crude extracts of *C. macowanii* leaves and their bacterial endophytes.

Compound name	Rt (min/sec)	*m*/*z*	Molecular formula	Reported biological activity	Sample
Melicopicine	5.64	330.1351	C_18_H_19_N_1_O_5_	Antiplasmodial activity [94]	T1
Lycorine	1.92; 13.76; 36.75; 7.23	288.1236	C_16_H_17_N_1_O_4_	Antibacterial cytotoxic and antitumor activities [95]	T1, T7, T9, T10
Angustine	10.35; 19.23; 25.98; 11.07	314.1388	C_20_H_15_N_3_O_1_	Antiproliferative activity [96]	T1, T7, T9, T10
3-O-methyl epimacowine	5.63	288.1586	C_17_H_21_NO_3_	Anticancer [97]	T7
Crinamidine	17.57; 15.50; 15.44; 6.32	318.1348	C_17_H_19_N_1_O_5_	Antibacterial [21] anticancer activity [98]	T1, T7, T9, T10
Vasicinol	4.18; 21.69; 21.51; 8.91	205.0977	C_11_H_12_N_2_O_2_	Antibacterial activity [99]	T1, T7, T9, T10
Aulicine	1.99	320.1870	C_18_H_25_NO_4_	Anticancer activity [97]	T9
Powelline	19.14, 14.05, 17.98, 7.43	302.1389	C_17_H_19_N_1_O_4_	Antitumor [100] antibacterial [21]	T1, T7, T9, T10
Brefeldin A	11.52	281.1702	C_16_H_24_O_4_	Anticancer [101] and antifungal [102]	T10

Note: T1 = *C. macowanii* leaves, T7 = *Bacillus safensis*, T9 = *Pseudomonas cichorii*, T10 = *Arthrobacter pascens*.

## Data Availability

All the data are provided in full in the results section of this paper apart from the DNA sequences of the bacterial endophytes available at https://www.ncbi.nlm.nih.gov/genbank, and accession numbers for each endophyte can be found in Table 1 of the manuscript.
